# Multiple Clinical Applications of a Dialkylcarbamoyl Chloride (DACC)-Coated Dressing in the Management of an Extensive Skin Defect: A Case Report

**DOI:** 10.7759/cureus.117539

**Published:** 2026-10-06

**Authors:** Yuki Miura, Junya Oshima, Kaoru Sasaki, Mitsuru Sekido

**Affiliations:** 1 Department of Plastic, Reconstructive, and Hand Surgery, University of Tsukuba, Ibaraki, JPN

**Keywords:** cadexomer iodine, dacc-coated dressing, dialkylcarbamoyl chloride, negative pressure wound therapy, skin grafting, wound management

## Abstract

Dialkylcarbamoyl chloride (DACC)-coated dressings physically bind and remove microorganisms through hydrophobic interactions and are widely used for various types of wounds, ranging from acute to chronic. However, few reports have comprehensively described their practical applications across different stages of wound management. We report the case of an 86-year-old woman with extensive skin necrosis of the lower extremity in whom DACC-coated dressings were used for four distinct purposes: as a standalone dressing, as a contact layer during negative pressure wound therapy (NPWT), as a contact layer for a skin graft, and as an interface with cadexomer iodine ointment. After debridement, a Sorbact^®^ Gel (ABIGO Medical AB, Askersund, Sweden) dressing maintained a moist wound environment and facilitated healthy granulation without clinical signs of infection. During NPWT, a Sorbact^®^ Compress was used as a contact layer, resulting in a wound bed suitable for skin grafting without clinical infection. A Sorbact^®^ Gel dressing was subsequently used as a contact layer over the skin graft, which achieved satisfactory engraftment. For the remaining highly exudative wound, a Sorbact^®^Compress was used in combination with cadexomer iodine ointment, providing adequate exudate management while preventing direct adherence of the ointment to the wound surface and pain during dressing changes. The wound ultimately healed without clinical infection, with a favorable outcome at seven months after skin grafting. This case illustrates the versatility of DACC-coated dressings and their potential to serve different therapeutic purposes according to the requirements of each stage of wound management.

## Introduction

In wound management, local treatment strategies are selected according to the condition of the wound and the therapeutic goals. Because these goals change throughout the wound-healing process, wound dressings are required to fulfill various roles at different stages, including infection control, wound protection, and exudate management.

Dialkylcarbamoyl chloride (DACC)-coated dressings (Sorbact®; ABIGO Medical AB, Askersund, Sweden) have a unique mechanism of action in which microorganisms bind to the dressing through hydrophobic interactions. This physical binding mechanism allows microorganisms to be captured and removed without killing them through antimicrobial action, thereby avoiding the risks of cytotoxicity and bacterial resistance [1,2]. Owing to these characteristics, DACC-coated dressings have been used for a wide range of wounds, including acute, chronic, and infected wounds [1].

Previous reports have described specific clinical applications of DACC-coated dressings, including their use as a primary dressing for cultured epithelial autografts [3] and as a wound contact layer during negative pressure wound therapy (NPWT) [4,5]. However, few reports have comprehensively described multiple clinical applications of DACC-coated dressings throughout the treatment course of a single patient.

In the present case, we used DACC-coated dressings for four distinct purposes in a patient with an extensive skin defect: as a standalone dressing, as a contact layer during negative pressure wound therapy (NPWT), as a contact layer for skin grafting, and as an interface with cadexomer iodine ointment. This report describes the clinical applications of DACC-coated dressings throughout the treatment course of a single patient and discusses their practical use in wound management.

## Case presentation

An 86-year-old woman sustained a crush injury after being trapped between two passenger vehicles and was transported to a local hospital. She was diagnosed with distal fractures of the right tibia and fibula and a crush injury of the right lower extremity. External fixation was performed on the day of injury. Subsequently, progressive skin necrosis developed, and she was transferred to our hospital 39 days after the injury with a diagnosis of skin necrosis of the right lower extremity.

On admission (hospital day (HD) 1), extensive skin and soft tissue necrosis was observed along the medial aspect of the thigh, knee, and lower leg. Although there was no purulent drainage, the granulation tissue appeared diffusely edematous. Plain radiographs of the lower extremity showed no osteolytic changes in the affected area. Contrast-enhanced computed tomography revealed no abscess formation but demonstrated edema extending to the level of the fascia. Because the fracture site was separate from the area of skin necrosis, flap coverage was considered unnecessary. We therefore planned initial debridement followed by delayed skin grafting.

On HD 5, the external fixator was removed, open reduction and internal fixation were performed, and extensive debridement was carried out under general anesthesia. The necrotic tissue was debrided extensively down to the fascia. Postoperatively, the wound was covered with a Sorbact® Gel dressing and gauze, with dressing changes performed every other day (Figure 1). Although heavy exudate and wound contamination were observed, healthy granulation tissue developed.

**Figure 1:**
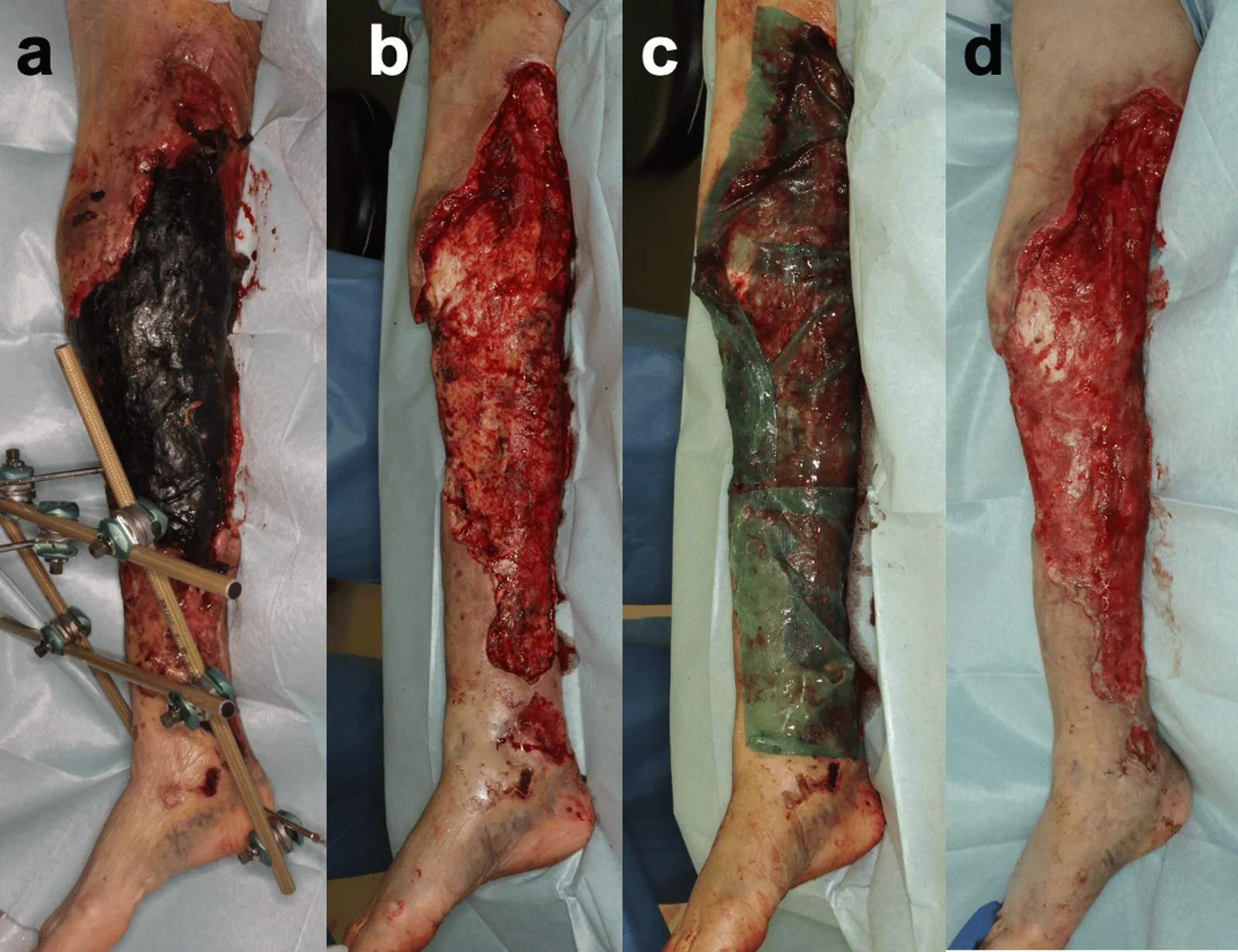
Standalone dressing (a) HD 1, on admission. Extensive black skin necrosis extending from the medial distal thigh to the distal lower leg. (b) HD 5. Debridement of the necrotic tissue. (c) HD 5. The wound was covered with a Sorbact® Gel dressing after debridement. (d) HD 11, six days after debridement. No residual necrotic tissue was observed, and extensive healthy granulation tissue had developed. HD: hospital day

Therefore, NPWT was initiated on HD 11 at a negative pressure of 80 mmHg, using a Sorbact® Compress as a contact layer (Figure 2). The dressings were changed every two to three days. Although heavy exudate and contamination extended to the superficial layers of the cotton filler, the wound bed continued to show healthy granulation tissue. A wound swab culture obtained on HD 27 was negative.

**Figure 2:**
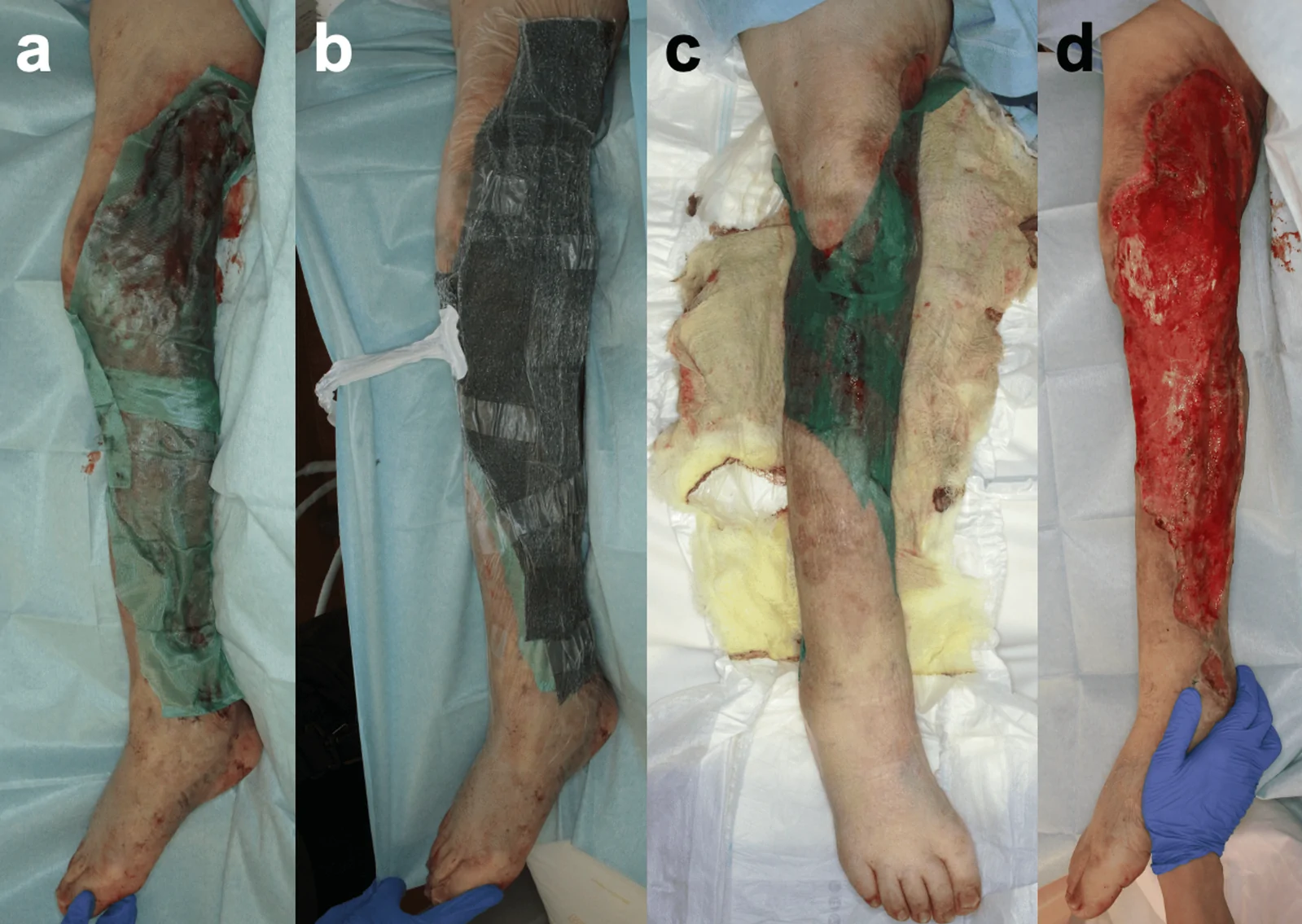
NPWT contact layer (a) HD 11, six days after debridement. Because healthy granulation tissue had developed, NPWT was initiated using a Sorbact® Compress as a contact layer. (b) A foam filler was placed over the Sorbact® Compress, and NPWT was initiated. (c) HD 19, eight days after initiation of NPWT. Substantial exudate and contamination were observed at the dressing change. (d) HD 19. After removal of the Sorbact® Compress, healthy granulation tissue was observed throughout the wound bed. HD: hospital day; NPWT: negative pressure wound therapy

On HD 40, split-thickness skin grafting using a 6:1 meshed graft was performed. At that time, the skin defect measured approximately 45 cm in length and 25 cm in width, with an estimated wound surface area of 630 cm². After the graft was secured with staples, a Sorbact® Gel dressing was placed directly over the graft as a contact layer and covered with cotton and gauze moistened with normal saline. Cefazolin was administered at a dose of 3 g/day for 14 days, starting on HD 40. The first dressing change was performed on HD 46 (Figure 3). Heavy yellow purulent-appearing exudate was observed; however, the graft showed no clinical signs of infection, such as erythema or increased local warmth. A wound swab culture grew *Pseudomonas aeruginosa* at a low bacterial burden of 10²-10³ CFU/mL.

**Figure 3:**
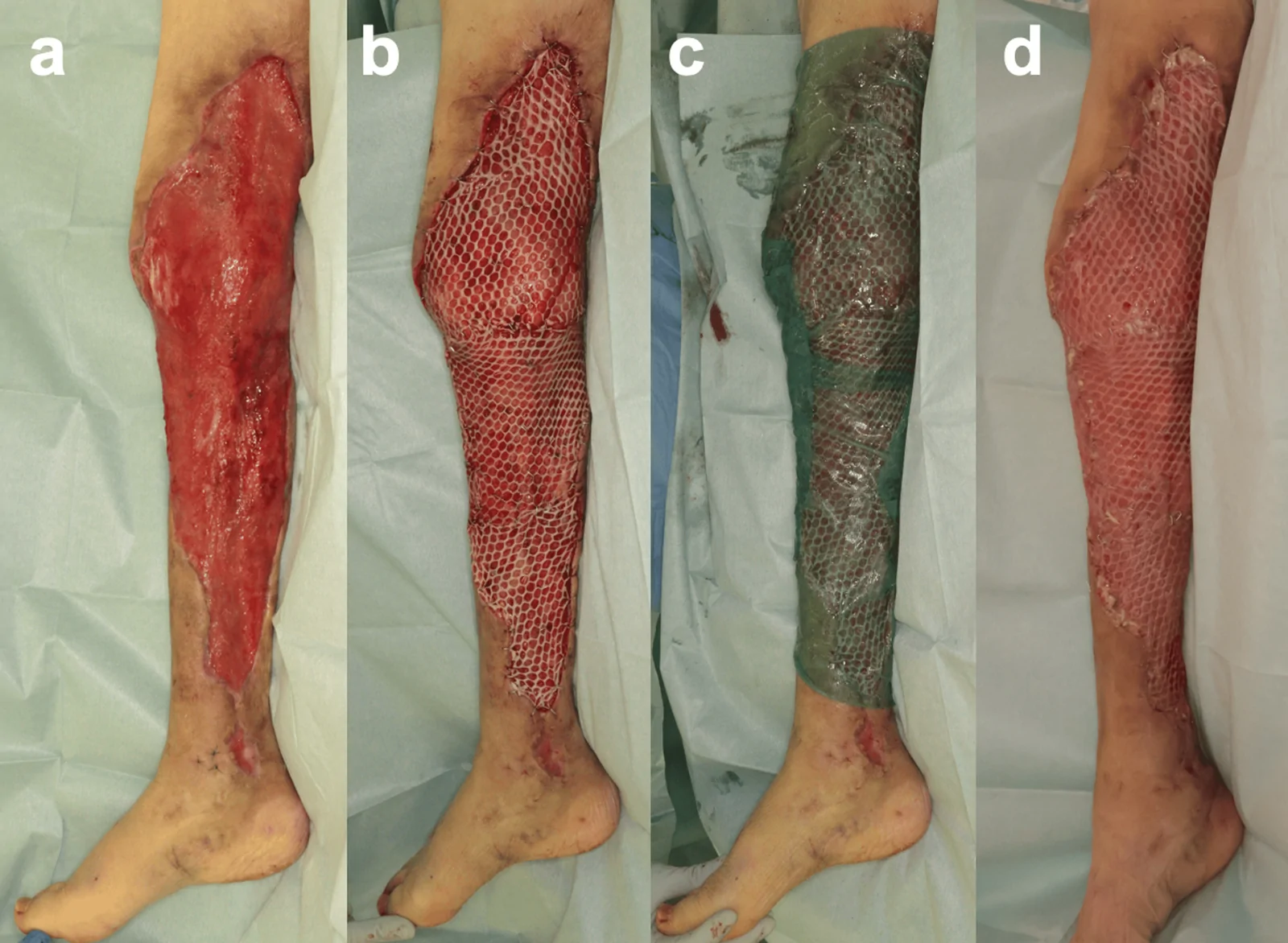
Skin graft contact layer (a) HD 40, immediately before skin grafting. The wound bed was covered with healthy granulation tissue. (b) The wound was covered with a 6:1 meshed skin graft. (c) A Sorbact® gel dressing was applied over the skin graft as a contact layer. (d) HD 46, six days after skin grafting. At the first postoperative dressing change, satisfactory graft take was observed. HD: hospital day

By HD 58, the lower leg had largely epithelialized, whereas epithelialization of the thigh remained incomplete, with edematous granulation tissue and heavy exudate. The dressing regimen was therefore changed to a Sorbact® Compress covered with cadexomer iodine ointment and gauze. At subsequent dressing changes, the cadexomer iodine ointment remained on the surface of the Sorbact® Compress without adhering directly to the grafted wound surface. No pain was reported during dressing removal.

By HD 71, the wound had largely epithelialized, and the patient was transferred to another hospital. At seven months after skin grafting, the grafted area showed no hypertrophic changes, and the wound remained in good condition (Figure 4).

**Figure 4:**
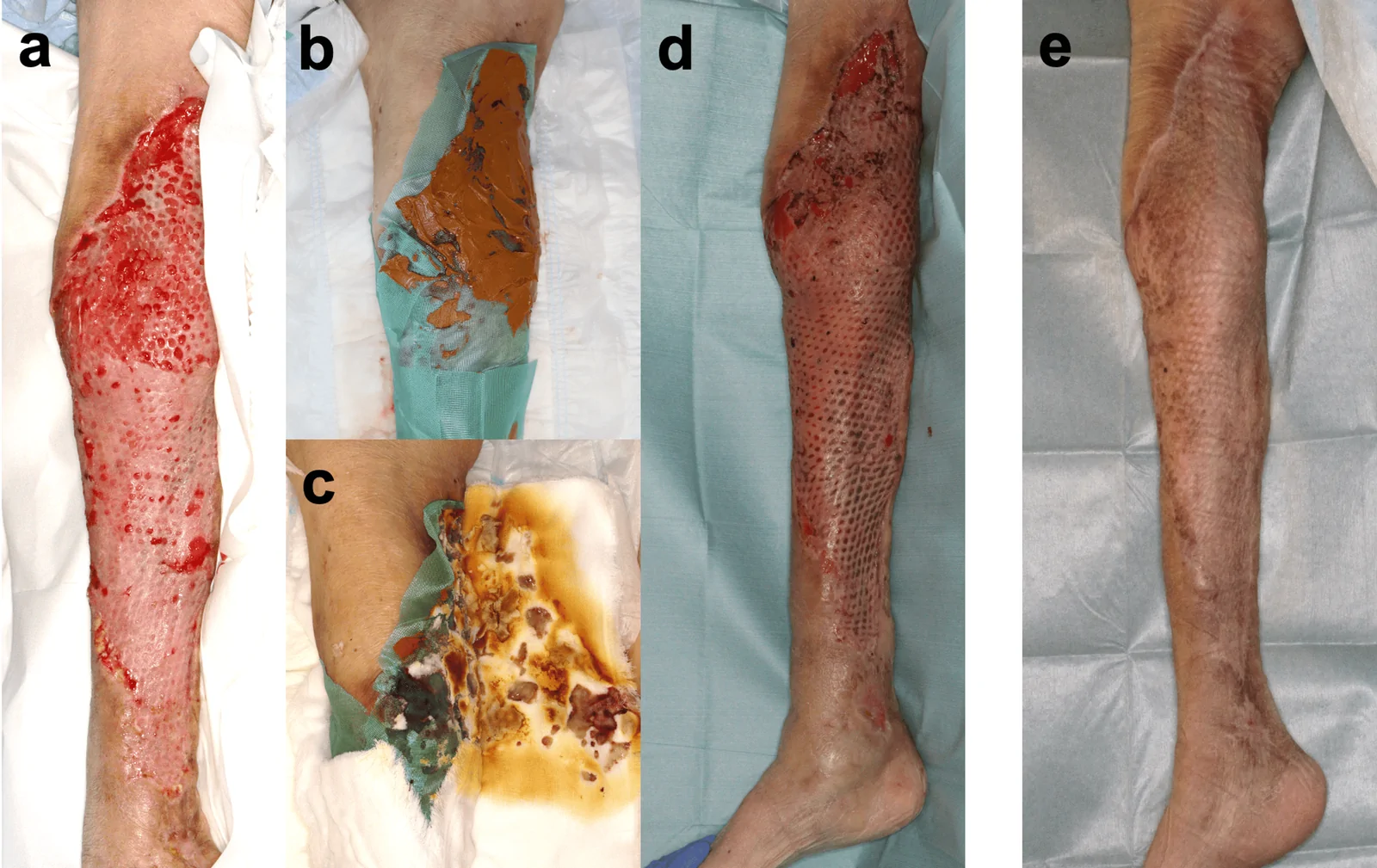
Cadexomer iodine interface (a) HD 57, 17 days after skin grafting. The lower leg had largely epithelialized, whereas edematous granulation tissue persisted in the thigh. (b) HD 58. Cadexomer iodine ointment was applied over the Sorbact® Compress, followed by gauze coverage. (c) HD 59, at the dressing change on the following day. The cadexomer iodine ointment had absorbed the wound exudate while remaining over the Sorbact® Compress. (d) HD 71. The wound had largely epithelialized at the time of transfer to another hospital. (e) Seven months after skin grafting. Epithelialization was maintained without scar contracture.

## Discussion

DACC-coated dressings are green mesh wound dressings composed of cellulose acetate coated with dialkylcarbamoyl chloride (DACC). DACC is a highly hydrophobic fatty acid derivative. Many microorganisms associated with wound colonization and infection have hydrophobic cell surfaces and therefore bind irreversibly to the DACC-coated dressing through hydrophobic interactions. These microorganisms, including *Staphylococcus (S.) aureus* (including methicillin-resistant *S. aureus* (MRSA)), Streptococcus species, *Escherichia coli*, *Pseudomonas aeruginosa*, and *Candida albicans*, are physically removed from the wound during dressing changes, thereby reducing the microbial burden at the wound site [1,2]. Because this mechanism relies on physical binding and removal rather than on the release of antimicrobial substances, DACC-coated dressings are considered unlikely to promote antimicrobial resistance and have minimal adverse effects on host cells, including fibroblasts and keratinocytes [2,3].

The roles required from local wound therapy vary according to the condition of the wound and the stage of wound healing. Therefore, wound dressings should be selected according to specific therapeutic goals such as infection control, wound protection, maintenance of an appropriate moist environment, and protection and fixation of skin grafts. DACC-coated dressings may potentially address these different therapeutic needs through modifications in their application while retaining their fundamental mechanism of microbial binding.

Previous reports on DACC-coated dressings have primarily focused on specific wound types or individual applications [3-5]. Few reports have examined their roles from the perspective of the changing therapeutic requirements across different stages of wound management. In this report, we describe the clinical applications of DACC-coated dressings from this perspective and discuss their potential utility in wound management.

In the present case, DACC-coated dressings were used for four distinct purposes: as a standalone dressing, as a contact layer during NPWT, as a contact layer for skin grafting, and as an interface with cadexomer iodine ointment.

Standalone dressing

The primary purpose of using a DACC-coated dressing as a standalone dressing is to reduce the wound bioburden by binding and removing microorganisms from the wound surface, thereby providing an environment favorable for wound healing.

DACC-coated dressings can remain in place for up to seven days while maintaining their effectiveness, potentially reducing the frequency of dressing changes and the associated burden on patients [4]. Different types of DACC-coated dressings can be selected according to the condition of the wound. The Sorbact® Gel dressing incorporates a hydrogel that provides a moist wound environment, which facilitates the irreversible binding of microorganisms to the dressing. Its flexibility also allows close contact with the wound surface and facilitates atraumatic removal [6].

In our practice, the Sorbact® Compress is generally used for wounds with moderate to high levels of exudate, whereas the Sorbact® Gel dressing is selected for dry or minimally exudative wounds because of its hydrogel component. In the present case, substantial wound contamination remained after debridement, making control of the wound bioburden an important consideration. In addition, maintenance of a moist wound environment was considered necessary to promote the development of a wound bed suitable for subsequent skin grafting. Therefore, the Sorbact® Gel dressing was selected for initial wound management.

NPWT contact layer

Previous studies have reported that bacterial counts in the wound may increase during NPWT, depending on the bacterial species [7]. In addition, direct contact between the wound surface and NPWT foam may result in adherence of the foam to the wound bed. The use of a DACC-coated dressing as a contact layer during NPWT may therefore offer two potential benefits: reduction of the wound bioburden and prevention of direct adherence between the NPWT foam and the wound surface.

Bateman used a DACC-coated dressing as a contact layer during NPWT in 10 patients with infected, exudative wounds. The exudate levels decreased within two weeks, and the wound cultures became negative in 60% (6/10) of the patients after one week and in all the patients after two weeks [4]. In another study involving 50 pressure ulcers, the use of a DACC-coated dressing as a contact layer during NPWT significantly reduced the bacterial burden at 48 hours and at seven days in moderately to heavily contaminated wounds with bacterial counts of ≥10⁴ CFU/mL [5]. Furthermore, interposing a DACC-coated dressing between the wound and NPWT foam may prevent adherence of the foam to the wound bed or exposed bowel, thereby reducing tissue injury [8]. This may also reduce pain during dressing changes.

In the present case, the wound culture was negative after 16 days of NPWT (HD 27), and a healthy wound bed suitable for skin grafting was achieved.

Skin graft contact layer

Paraffin gauze or silicone mesh dressings are commonly used as contact layers immediately after skin grafting, with gauze or cotton dressings placed over them for protection and fixation [9]. However, conventional paraffin gauze and silicone mesh dressings lack the ability to physically bind and remove microorganisms, unlike DACC-coated dressings.

Nakamura et al. divided split-thickness skin graft sites into two areas and applied the Sorbact® Compress to one area and silicone gauze to the other as contact layers. At seven days after surgery, the bacterial count was significantly lower in the area treated with the Sorbact® Compress (P = 0.0175), while no significant difference in graft take was observed between the two groups [10]. Choi et al. used Sorbact® gel dressing as a contact layer after skin grafting in seven patients and reported no infections in any of the cases. They suggested that the dressing provided both a bacterial burden reduction and moisture-retaining properties, which are not provided by conventional petrolatum or paraffin gauze dressings alone [11].

In the present case, Sorbact® Gel dressing was used as a contact layer over the skin graft. At the first dressing change approximately one week after grafting, no clinical signs of infection were observed, and wound culture demonstrated only a low bacterial burden.

Cadexomer iodine interface

In highly exudative wounds, DACC-coated dressings alone may not provide sufficient exudate management. The combination of a DACC-coated dressing with cadexomer iodine ointment may therefore help manage excessive exudate while retaining the potential benefit of reducing the wound bioburden.

To our knowledge, no previous reports have described the combined use of DACC-coated dressings and cadexomer iodine ointment. The manufacturer's instructions for use state that DACC-coated dressings should not be used in combination with greasy ointments, creams, oxidizing agents, or hydrophobic liquids [12]. Although cadexomer iodine ointment does not contain a hydrophobic ointment base, its compatibility with DACC-coated dressings has not been established. Cadexomer iodine ointment has a high fluid-absorption capacity, with its water absorption at 24 hours reported to be 2.9 times that of povidone-iodine sugar ointment [13]. Interposing a DACC-coated dressing between the wound surface and cadexomer iodine ointment may allow exudate management while preventing direct adherence of the ointment to the wound, potentially reducing pain and the burden associated with dressing changes. However, whether both dressings retain their respective properties when used in combination remains unclear.

In the present case, edematous granulation tissue and excessive exudate persisted in the thigh wound 17 days after skin grafting (HD 57), and the dressing regimen was therefore changed to a combination of a Sorbact® Compress and cadexomer iodine ointment. Subsequently, the exudate decreased and epithelialization progressed. The cadexomer iodine ointment did not adhere directly to the wound surface, and no pain was reported during dressing changes.

Figure 5 summarizes the applications of DACC-coated dressings used in the present case. Although these represent only some of their potential applications, the ability to use a single type of dressing for different purposes may be an important advantage of DACC-coated dressings. In wound management, dressings should be selected not only according to the type of wound but also according to the therapeutic goals at each stage of wound healing. The versatility of DACC-coated dressings may allow them to fulfill different roles as these therapeutic goals change.

**Figure 5:**
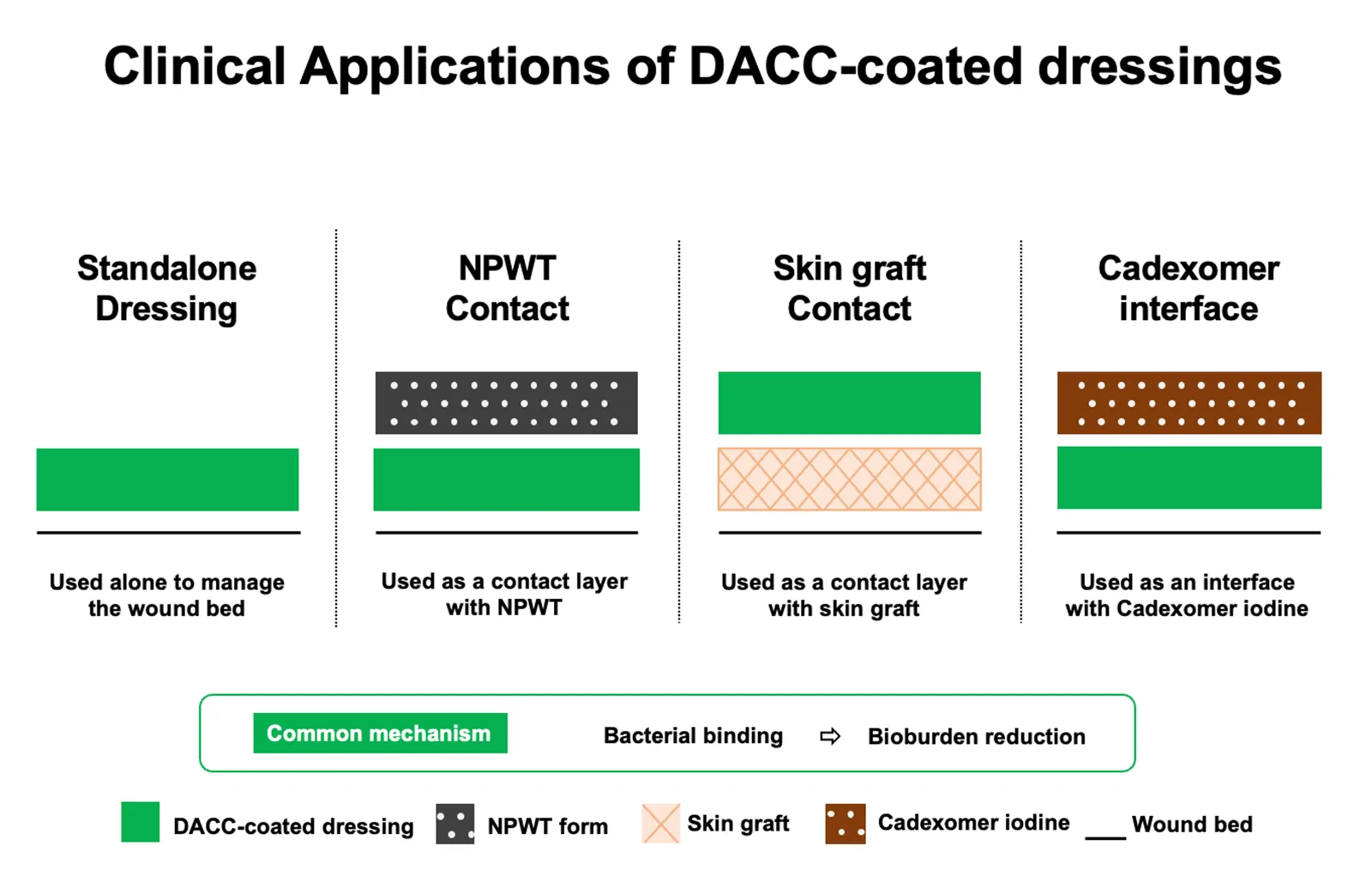
Clinical applications of DACC-coated dressings in wound management Dialkylcarbamoyl chloride (DACC)-coated dressings reduce the wound bioburden by binding and removing microorganisms. In the present case, DACC-coated dressings were used for four distinct purposes according to the therapeutic requirements at each stage of wound management: as a standalone dressing, as a contact layer during negative pressure wound therapy (NPWT), as a contact layer for skin grafting, and as an interface with cadexomer iodine ointment. This schematic was created by the authors using Microsoft PowerPoint (Microsoft Corporation, Redmond, WA, USA). No generative artificial intelligence tools were used.

However, this report describes a single case in which other treatments, including NPWT, skin grafting, systemic antibiotics, and cadexomer iodine ointment, were also used at different stages of wound management. Therefore, the therapeutic effects of DACC-coated dressings alone cannot be determined. Further studies are needed to evaluate the efficacy of each application of DACC-coated dressings and their use in combination with other wound treatments. 

## Conclusions

In this case of an extensive skin defect, DACC-coated dressings were used for four distinct purposes: as a standalone dressing, as a contact layer during NPWT, as a contact layer for skin grafting, and as an interface with cadexomer iodine ointment. Wound management was completed without clinically apparent infection. These different applications allowed the same type of dressing to be incorporated into wound management according to the therapeutic requirements at each stage.

DACC-coated dressings may represent a versatile option throughout the wound-healing process through the selection of different dressing formulations and their use in combination with other treatment modalities such as NPWT and absorbent topical agents. This case illustrates the flexibility of DACC-coated dressings in addressing different therapeutic requirements at various stages of wound management while retaining their fundamental mechanism of microbial binding.
